# SATB1 plays an oncogenic role in esophageal cancer by up-regulation of FN1 and PDGFRB

**DOI:** 10.18632/oncotarget.14849

**Published:** 2017-01-27

**Authors:** Guiqin Song, Kang Liu, Xiaolin Yang, Bo Mu, Junbao Yang, Lang He, Xin Hu, Qiujiang Li, Yunxia Zhao, Xiaoming Cai, Gang Feng

**Affiliations:** ^1^ Department of Biology, North Sichuan Medical College, Nanchong, Sichuan, P.R. China; ^2^ Institute of Tissue Engineering and Stem Cells, The Second Clinical Medical College of North Sichuan Medical College, Nanchong Central Hospital, Nanchong, Sichuan, P.R. China; ^3^ Biotherapy Center, Nanchong Central Hospital, Nanchong, Sichuan, P.R. China; ^4^ Clinical college of North Sichuan Medical College, Nanchong, Sichuan, P.R. China; ^5^ State Key Laboratory of Biotherapy, Sichuan University, Chengdu, Sichuan, P.R. China

**Keywords:** SATB1, esophageal cancer, FN1, PDGFRB

## Abstract

Esophageal cancer is a highly aggressive malignancy with very poor overall prognosis. Given the strong clinical relevance of SATB1 in esophagus cancer and other cancers suggested by previous studies, the exact function of SATB1 in esophagus cancer development is still unknown. Here we showed that the knockdown of SATB1 in esophageal cancer cell lines diminished the cell proliferation, survival and invasion. Whole genome transcriptome analysis of SATB1 knockdown cells revealed the different gene expression profiles between TE-1 cells and MDA-MB-231 cells. Network analysis and functional experiments further identified FN1 and PDGFRB to be key downstream genes regulated by SATB1 in esophageal cancer cells. Importantly, FN1 and PDGFRB were found to be highly expressed in human esophageal cancer. In summary, we provided the first molecular evidence that SATB1 played an oncogenic role in esophageal cancer by up-regulation of FN1 and PDGFRB.

## INTRODUCTION

Esophageal cancer (or oesophageal cancer) is one of the most aggressive malignancies and its morbidity is still on the rise with very poor overall survival rate [1–3]. The overall 5-year survival rate reported was only around 20% [4]. The estimated incidence for men is three times more than for women [3]. Despite recent clinical advances in esophageal cancer, the overall patient's prognosis remains poor. There is still a lot need to be defined, including effective screening, diagnosis and management strategy of esophageal cancer [1].

Special AT-rich sequence-binding protein 1 (SATB1), a global chromatin organizer and transcription factor, involved in chromatin ‘loopscape’ organization and response to physiological stimuli [5]. It is already known that SATB1 is an oncogene which promotes breast tumor growth and metastasis [6]; its expression was reported in several breast cancer cell lines and tumor biopsies [7–9]. The association of SATB1 was also observed in several other cancers [9], including colorectal cancer [10–12], prostate cancer [13, 14], endometriod endometrial cancer [15, 16], liver cancer [17], rectal cancer [18], bladder cancer [19], ovarian cancer [20] and gastric cancer [21].

While in radically resected upper gastrointestinal tract adenocarcinoma the overexpression of SATB1 correlates metastases, shorter overall survival as well as with shorter recurrence-free survival [22]. Cong *et al*. [2015] also found esophageal squamous cell carcinoma (ESCC) patients with high SATB1 expression had significantly shorter survival than those with low SATB1 expression, which indicates high SATB1 expression might serve as a predictive biomarker of poor prognosis in ESCC and possibly could be a promising new candidate for targeted therapies [23]. Given the strong clinical relevance, the exact function of SATB1 in esophagus cancer development is still unknown.

The present study was carried out to explore the function of SATB1 in the development of human esophagus cancer. We evaluated the impact of SATB1 knockdown on cell proliferation, survival, apoptosis and migration. We demonstrated that SATB1 functions differently in esophagus cancer versus breast cancer by comparing their gene expression profiles. The function of two SATB1 major downstream genes, FN1 and PDGFRB, was also discussed. It is of great importance to elucidate the molecular pathology in order to improve the prognosis for esophageal cancer patients.

## RESULTS

### SATB1 promoted TE-1 cell survival and migration

**Figure 1:**
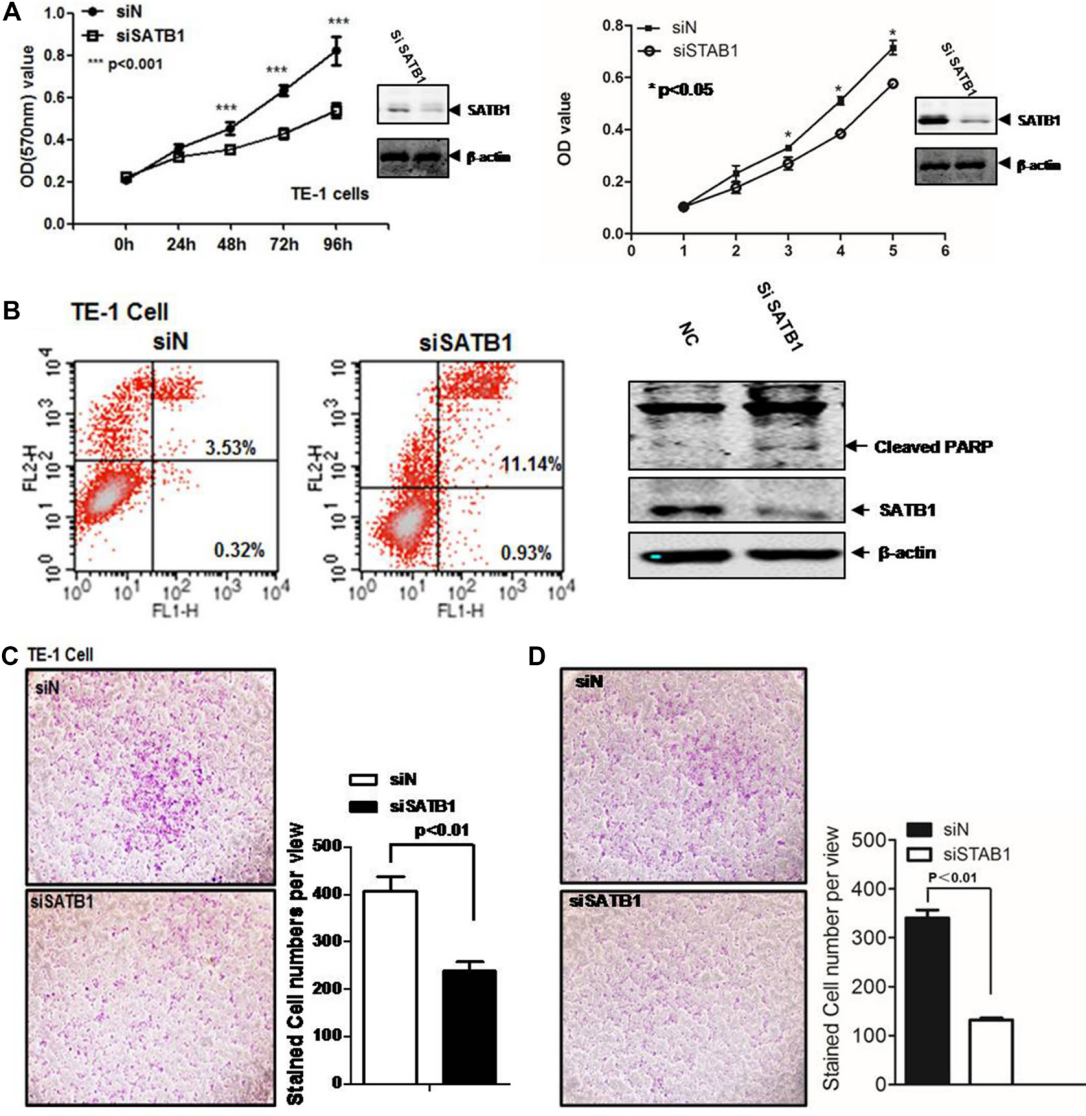
SATB1 promotes TE-1 and EC-109 cell survival and migration (**A**) MTT is employed to measure the cell viability in TE-1 and EC-109 cells. siN is the siRNA pool for control and siSATB1 is siRNA pool for SATB1; (**B**) Flow cytometry was performed to analyze the cell apoptosis. FL1-H is annexin V and FL2-H is PI. Western blot was performed to detect the cleaved PARP. Cell invasion/migration was evaluated by Transwell assays for (**C**) TE-1 cells and (**D**) EC-109 cells. The results are the mean ± SEM of three independent experiments.

To explore the molecular function of SATB1 in esophageal cancer, we evaluated the cell viability using MTT assay. The endogenous expression level of SATB1 was first checked in two esophageal cancer cell lines, TE-1 and EC109. As showed in Supplementary Figure 1, SATB1 was expressed in both cells. The MTT assay was then performed for both cells. Aliquots of early log phase 5 × 10^3^/well SATB1 knockdown cells (siSATB1) or control cells (siN) were cultured in 96-well plates for 0, 24, 48, and 72 hrs. The absorbance value of the MTT converted dye for each time point was showed in Figure 1A (TE-1 left panel; EC-109, right panel). The knockdown of SATB1 diminished the proliferation and survival of TE-1 cells from 48 h to 96 h (*p* < 0.001). Similarly, a minor but statistical significant reduction was also observed in EC-109 cells (*p* < 0.05). Spontaneous apoptosis in TE-1 cells was assessed by FACS analysis of Annexin-V and propidium iodide (PI) staining (Figure 1B). The SATB1 knockdown indeed caused increased apoptosis in TE-1 cells from 3.87% to 12.07%. PI staining revealed that the majority was in the late apoptotic stage (3.53% vs 11.14%). Increased cleaved PARP was found in TE-1 SATB1 knockdown cells (Figure 1B, right panel). Similar results were also obtained for EC-109 SATB1 knockdown cells (Supplementary Figure 2).

Cell motility is critical for esophageal cancer metastasis. The impact of SATB1 expression on the invasion/migration capability in TE-1 or EC-109 cells was evaluated by the Transwell assay. As showed in Figure 1C and Figure 1D, the knockdown of STAB1 by siRNA in these two cell lines was able to induce anti-invasive effects *in vitro*. Down-regulation of SATB1 inhibited the cells migration to bottom chambers by around 40%, respectively (Figure 1C and 1D).

### Whole genome transcriptome analysis identified the downstream genes of SATB1 in TE-1 cells

**Figure 2:**
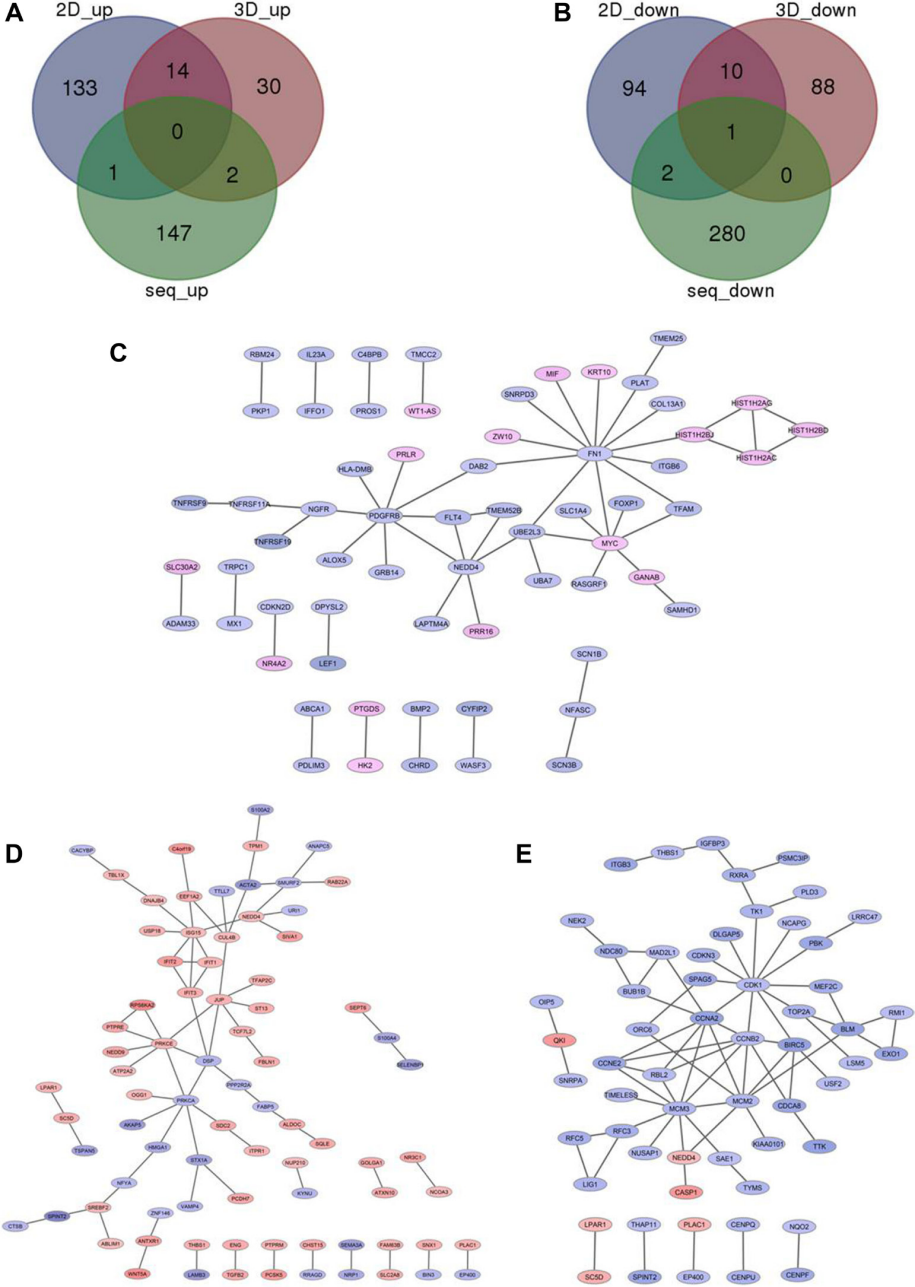
Overlapping the down-regulated genes (**A**) and up-regulated genes (**B**) after knock-down of SATB1 in TE-1 cells (green part) or MDA-MB-231 cells under 2D (blue part) or 3D culture (red part). PPI network analysis those significantly changed genes after knock-down of SATB1 in TE-1 cells (**C**) or MDA-MB-231 cells under 2D (**D**) or 3D culture (**E**).

Under the condition of |log_2_(fold change)|> 0.5 and adjusted *p value* < 0.05, 433 differentially expressed genes (DEGs) were identified in Comparison 1 (siSATB1 vs siControl in TE-1 cells), among which 150 genes were up-regulated (Supplementary Figure 3, red part and Figure 2A, green part) and 283 were down-regulated (Supplementary Figure 3, green part, and Figure 2B, green part). Given that SATB1 is an oncogene which promotes breast tumor growth and metastasis [6], we were wondering if the downstream genes regulated by SATB1 are similar between esophageal cancer cells and breast cancer cells. Therefore, similar analyses were also performed to identify the differentially changed genes in breast cancer cells after knock-down of SATB1 [6]. 255 DEGs were identified for Comparison 2 (shSATB1 vs shControl in MDA-MB-231cells under 2D culture condition), of which 148 were up-regulated (Figure 2A, blue part) and 107 were down-regulated (Figure 2B, blue part); 145 DEGs were identified for Comparison 3 (shSATB1 vs shControl in MDA-MB-231cells under 3D culture condition), among which 46 were up-regulated (Figure 2A, purple part) and 99 were down-regulated (Figure 2B, purple part) (Table 1, Supplementary Figure 3, Supplementary Tables 1 and 2).

**Table 1:** Significantly changed genes after knock-down of SATB1 in TE-1 cells or MDA-MB-231 cells under 2D or 3D culture

Items	Count of DEGs(|log_2_(fold change)| > 0.5 & p.adjust value < 0.05)	
RNA_seq(Comparison 1)	433	150 (up) 283 (down)
Array_2D(Comparison 2)	255	148 (up 107 (down)
Array_3D(Comparison 3)	145	46 (up) 99 (down)

**Table 2:** GO analysis ofsignificantly changed genes in SATB1knock-down TE-1 cells

ID	Description	p.adjust	Count
GO-0051179	localization	1.37E-03	125
GO-0065007	biological regulation	1.68E-03	212
GO-0050896	response to stimulus	1.75E-03	163
GO-0009987	cellular process	2.65E-03	262
GO-0023051	regulation of signaling	2.65E-03	75
GO-0009966	regulation of signal transduction	2.69E-03	69
GO-0016477	cell migration	5.20E-03	36

DEGs of these three comparisons were overlapped, and common DEGs and those genes existing only in two comparisons were identified (Supplementary Tables 1 and 2). Between Comparison 1 and Comparison 2, only 4 common DEGs identified: the common up-regulated DEG identified was DNA-Damage-Inducible Transcript 4 (DDIT4); common down-regulated DEGs were Protein Kinase (CAMP-Dependent, Catalytic) Inhibitor Alpha (PKIA), WAS/WASL Interacting Protein Family, Member 1 (WIPF1) and SATB1. Between Comparison 1 and Comparison 3, only 3 DEGs identified: the common up-regulated DEG was Secretory Leukocyte Peptidase Inhibitor (SLPI); common down-regulated DEGs were Apolipoprotein C-I (APOC1) and SATB1. In all three comparisons, SATB1 was the only common DEG which was downregulated. No other genes were found commonly regulated by SATB1 between TE-1 cells and MDA-MB-231cells, suggesting that the downstream genes or functions of SATB1 in different cancer cells might be different.

### Construction of biological networks analysis showed FN1 and PDGFRB were hub genes regulated by SATB1 in TE-1 cells

PPI networks were constructed and visualized in Cytoscape [24] for significantly changed genes after knock-down of SATB1 in TE-1 cells and MDA-MB-231 cells under 2D culture condition and 3D culture condition (Figure 2C, 2D and 2E). For Comparison 1, key genes were Fibronectin 1 (FN1), Platelet-Derived Growth Factor Receptor, Beta Polypeptide (PDGFRB), Neural Precursor Cell Expressed, Developmentally Down-Regulated 4 (NEDD4) and MYC. For Comparison 2, key genes were Ubiquitin-Like Modifier (ISG15), Protein Kinase C, Alpha (PRKCA), PRKCE (Protein Kinase C, Epsilon), Junction Plakoglobin (JUP) and Desmoplakin (DSP). For Comparison 3, key genes were Cyclin-Dependent Kinase 1 (CDK1), Minichromosome Maintenance Complex Component 3 (MCM3), Cyclin B2 (CCNB2), Cyclin A2 (CCNA2) and Minichromosome Maintenance Complex Component 2 (MCM2). No similar key genes were found between TE-1 and MDA-MB-231 cells, also indicating SATB1 conducted its oncogenic role in different type cancers by regulation of different genes.

### GO and KEGG pathway analysis showed FN1 and PDGFRB were key genes in the top changed pathways regulated by SATB1 in TE-1 cells

To gain better insight into the gene interactions that caused by SATB1 knockdown in cancer cells, corresponding GO biological process and KEGG pathway analysis were conducted for DEGs of each comparison.

**Figure 3:**
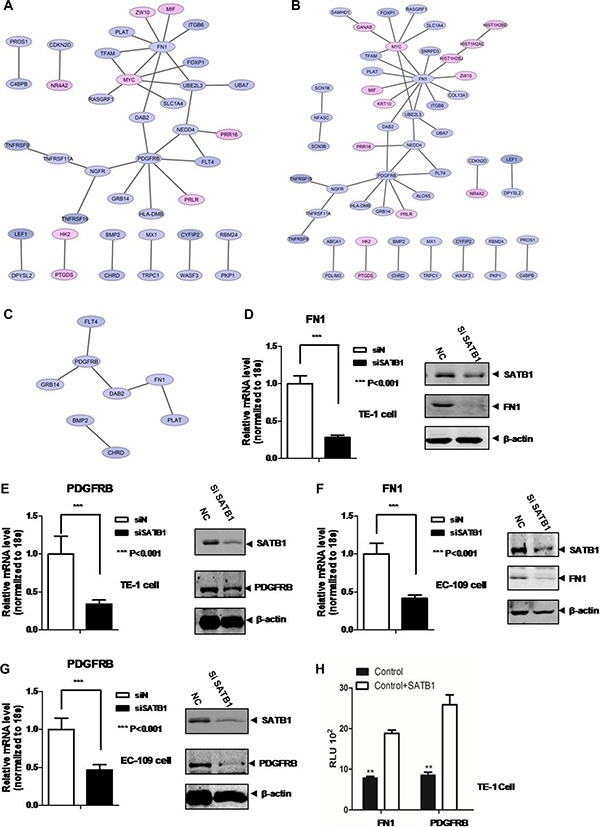
PPI network analysis of DEGs in (**A**) “biological regulation” , (**B**) “cellular process” , (**C**) “cell migration” pathways in TE-1 SATB1 knockdown cells. qRT-PCR and western blot were employed to validate (**D**) FN1 and (**E**) PDGFRB expression after knock-down of SATB1 in TE-1 cells. qRT-PCR and western blot were performed to validate FN1 (**F**) and PDGFRB (**G**) expression after knock-down of SATB1 in EC-109 cells, (**H**) Luciferease reporter assay.

For Comparison 1, significant GO biological pathways were “localization”, “biological regulation”, “response to stimulus”, “cellular process”, “regulation of signaling”, “regulation of signal transduction” and “cell migration” (Table 2). No KEGG for this comparison. PPI networks were also constructed for DEGs in “biological regulation” (Figure 3A), and “cellular process” (Figure 3B) and “cell migration” (Figure 3C) in SATB1 knock-down TE-1 cells. FN1 and PDGFRB were key genes for these pathways. For Comparison 2, significant GO biological pathways were “cell differentiation”, “biological regulation”, “apoptotic process”, “epithelium development”, “cell migration” and “response to stimulus” (Table 3). No KEGG pathway identified for this. For Comparison 3, significant GO biological processes were “cell cycle” and “cell division”; significant KEGG pathways included “cell cycle”, “DNA replication” and “p53 signaling pathway” (Table 4A and 4B).

**Table 3:** GO analysis ofsignificantly changed genes after knock-down of SATB1 in MDA-MB-231 cells under 2D culture

ID	Description	p.adjust	Count
GO-0030154	cell differentiation	1.27E-07	88
GO-0065007	biological regulation	1.11E-06	187
GO-0006915	apoptotic process	2.41E-05	50
GO-0060429	epithelium development	2.41E-05	36
GO-0016477	cell migration	4.28E-05	36
GO-0050896	response to stimulus	4.99E-05	141

**Table 4A:** GO analysis of significantly changed genes after knock-down of SATB1 in MDA-MB-231 cells under 3D culture

ID	Description	p.adjust	Count
GO-0007049	cell cycle	1.55E-16	51
GO-0051301	cell division	4.76E-11	27

**Table 4B:** KEGG analysis of significantly changed genes after knock-down of SATB1 in MDA-MB-231 cells under 3D culture

ID	Description	p.adjust	Count
hsa04110	cell cycle	2.62E-06	11
hsa03030	DNA replication	6.43E-05	6
sa04115	p53 signaling pathway	1.30E-02	5

The majority of GO enriched pathways were different between TE-1 and MDA-MB-231 cells, further suggesting SATB1 has different regulatory function in esophageal cancer cells and breast cancer cells.

### FN1 and PDGFRB were positively regulated by SATB1 and played important roles in cell survival and migration

To explore the function of two key genes FN1 and PDGFRB in esophageal cancer cells, qRT-PCR and western blot were employed to detect the mRNA and protein expression level change of these two genes in SATB1 knockdown TE-1 and EC-109 cells. The results showed a significant ~4-fold reduction of FN1 mRNA level and ~3-fold reduction of PDGFRB mRNA level after SATB1 knockdown in TE-1 cells (Figure 3D and 3E, left). While the two target protein level after SATB1 knockdown was also greatly reduced (Figure 3D and 3E, right). Similarly, knockdown of SATB1 in EC-109 cells also caused mRNA and protein expression level reduction for both genes showed by qPCR and western blots (Figure 3F and 3G). The regulatory role of SATB1 was further demonstrated by the luciferase reporter assay. As showed in the Figure 3H, the luciferase signaling driven by the promoters of FN1 or PDGFRB was significantly increased (~2.5 fold) after SATB1 transfection.

**Figure 4:**
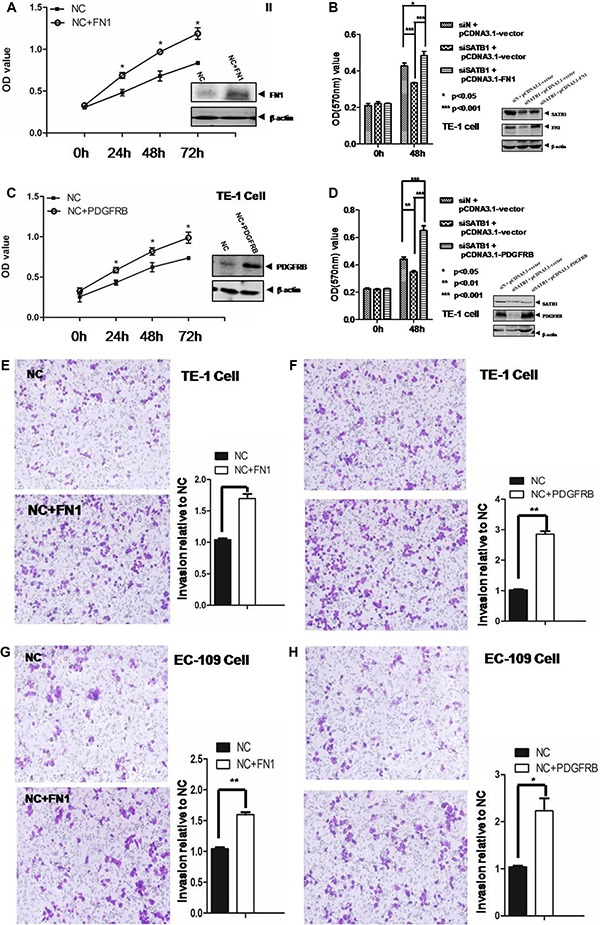
MTT assay was employed to measure the cell proliferation in TE-1 cells (**A**) after overexpression FN1. and (**B**) after knockdown of SATB1 or/and overexpression of FN1 and (**C**) after overexpression PDGFRB and (**D**) after knockdown of SATB1 or/and overexpression of PDGFRB. Cell invasion/migration was evaluated by Transwell assays for (**E**) TE-1 cells overexpression of FN1 and (**F**) TE-1 cells overexpression of PDGFRB and (**G**) EC-109 cells overexpression of FN1 and (**H**). EC-109 cells overexpression of PDGFRB. The results are the mean ± SEM of three independent experiments.

The impact of these two genes on cancer cell survival was further assessed by MTT assay. As showed in Figure 4A, the overexpression of FN1 significantly increased the proliferation and survival of TE-1 cells from 48 h to 96 h (*p* < 0.05). Similar trend was observed for the PDGFRB overexpression (*p* < 0.05) (Figure 4C). Similar results were observed in EC-109 cell overexpression of FN1 or PDGFRB (Supplementary Figure 4). While knockdown of SATB1 caused the reduced expression of FN1, this reduction was reversed by the overexpression of pcDNA3.1-FN1 (Figure 4B). The MTT readout indicated that the diminished proliferation and survival at 48 h in SATB1 knockdown TE-1 cells was reversed by the overexpression of FN1. The overexpression of FN1 in TE-1 cells showed an even statistically higher survival rate compared to mock treatment (Figure 4B). Similar trend was also observed for PDGFRB (Figure 4D). The overexpression of PDGFRB rescued the lower viability in SATB1 knockdown cells and showed an even higher proliferation rate compared to control group.

The Transwell assay was employed to evaluate the invasion/migration capability for these two genes. As showed in Figure 4E and 4F, the number of total cells migrated to the downside of membrane was significantly increased in FN-1 (1.6 folds) or PDGFRB (2.8 folds) over-expressed TE-1 cells. Similarly, a 1.5-fold and 2.2-fold increased invasion capability was observed in EC-109 cells with FN-1 or PDGFRB overexpression respectively (Figure 4G and 4H).

**Figure 5:**
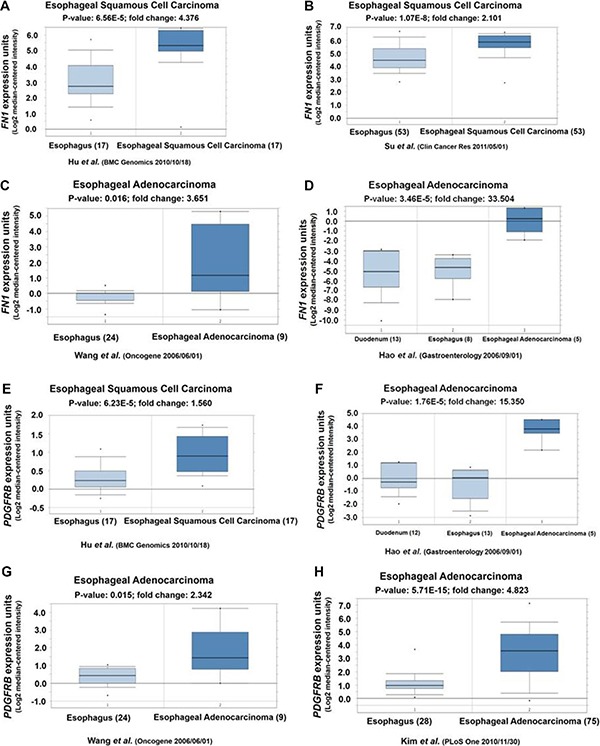
(**A**, **B**, **C** and **D**) Oncomine analyses showed FN1 highly expressed in human esophageal squamous cell carcinoma and esophageal adenocarcinoma. (**E**, **F**, **G**, and **H**) Oncomine analyses showed PDGFRB highly expressed in human esophageal squamous cell carcinoma and esophageal adenocarcinoma.

To further explore the clinical significance of FN1 and PDGFRB expression in human esophageal cancer patients, we performed the online database analysis (Oncomine) to examine their expressions in human esophageal cancer patients. As showed in Figure 5A–5D), a significant elevated 2.1~33.5–fold overexpression was observed for FN1. Similarly, PDGFRB was also overexpressed (1.5~15.4 folds) in esophageal squamous cell carcinoma and esophageal adenocarcinoma patients (Figure 5E–5H).

## DISCUSSION

Accumulated evidences suggested that STAB1 is an oncoprotein in various tumors; however, the exact function is still unknown in esophageal cancer. Our MTT results showed that the knockdown of SATB1 in highly expressed esophageal cancer cells diminished their proliferation and survival from 48 h to 72 h. Consistently, spontaneous apoptosis was increased when SATB1 was knockdown. As far as we know, this is the first molecular evidence suggesting the SATB1 function in promotion of esophageal cancer cell proliferation and survival. The early lymph node metastasis and invasion to neighboring organs is one of the major cause for the poor prognosis of esophageal cancer [25]. Understand the pathophysiological mechanism is of great clinical significance. Several genes/pathways have been implicated to esophageal cancer migration, such as Cdc42 [26], HGF/SF [27], Androgen receptor [28], RhoA, Rac-1, and Cdc42 [29], VEGF [30], HER2 [31], PLCE1 [32], ABCG2/V-ATPase, MMS19 [33]. However, the exact mechanism is still unknown. Here we showed that the migration of TE-1cells was inhibited by knockdown of STAB1 in *in vitro* Transwell assay, suggesting STAB1 is one of the contributors for esophageal cancer invasion. SATB1 overexpression outside the normal physiological context renders the cancer cells of a high metastatic potential.

SATB1 promotes breast tumor growth and metastasis [6]. It is unknown in esophageal cancer whether SATB1 has conserved or different function in regulation of the whole genomic transcriptome as in breast cancer. The overlapping analysis for DEGs of TE-1 and MDA-MB-231cells showed that there is no common gene identified in all three comparisons while only quite few common genes were found in two comparisons, suggesting STAB1 plays different regulatory roles in esophageal cancer development. This conclusion was further confirmed by the enriched GO biological network analysis. We did not find significant similarities between biological pathways or processes examined. Our results illustrated the unique functional regulatory role of SATB1 for the esophageal cancer whole genomic transcriptome. Interestingly, the comparisons between different culture conditions (2D vs 3D) of same breast cancer cells showed no common enriched pathways; suggesting different microenvironment also might play important roles in the gene regulation. As these two culture conditions differs in simulating important tumor characteristics like hypoxia, dormancy and anti-apoptotic features [34].

The “biological regulation”, “cellular process” and “cell migration” pathways enriched in SATB1 knockdown TE-1 cells all have two highly connected genes: FN1 and PDGFRB, implying these two hub genes might play essential roles in above mentioned pathways. Our results (Figure 3A–3H) indicated the expression level of FN1 and PDGFRB were upregulated by SATB1. The overexpression of FN1 or PDGFRB also enhanced the cell proliferation and migration ability (Figure 4A, 4C, 4E–4H), highlighting the importance of these two gene in esophageal cancer cells proliferation/invasion although the underlying mechanisms remains unknown. Possibly, like in some other cancer cells, FN1 induced specific matrix metalloproteinases expression, such as MMP9/MMP2, to promote invasion and metastasis [35–37]. PDGFRB was found could increase glioma stem cell growth and survival [38], mammary tumor cells migration [39], however, little is known about the PDGFRB signaling in esophageal cancer cells. To further dissect the molecular mechanism of STAB1 in esophageal cancer pathogenesis, these two downstream genes, FN1 and PDGFRB, were overexpressed and their impact on cell survival and migration was evaluated in SATB1 knockdown cells. The results suggested that overexpression of FN1 or PDGFRB not only can compensate the reduced survival rate in STAB1 knockdown cells, but can promote even higher cell proliferation rate. Consistent with the results we observed in current study, a literature survey indicated that the overexpression of these two genes associated with esophageal squamous cell carcinoma [40, 41] or esophageal adenocarcinoma [42–44] (Figure 5).

In conclusion, SATB1 is an oncogenic gene involved in esophageal cancer tumor cell proliferation and metastatic potential regulation; its function is partially delivered by the downstream genes FN1 and PDGFRB. SATB1 might serve as a therapeutic target and prognostic marker for esophageal cancer. Understanding the function of SATB1 in esophageal cancer could have potential implication for diagnosis and therapy.

## MATERIALS AND METHODS

### Tissue culture, siRNA transfection and antibodies

Two esophageal cancer cell lines, TE-1 and EC-109, were purchased from American Type Culture Collection (ATCC, Manassas VA) and maintained in RPMI-1640 supplemented with 10% fetal bovine serum, 100 units/mL penicillin, 100 μg/mL streptomycin in a humidified tissue culture incubator with 5% CO2 at 37°C.

Expression vectors of pcDNA3.1-FN1 and pcDNA3.1- PDGFRB were cloned from the cDNA sequences purchased from Dharmacon. Transfection of TE-1 and EC-109 cells was carried out using *Lipofectamine*^®^ *2000* reagent (Invitrogen, CA) according to manufacturer's instructions.

Antibodies used in this study were: SATB1 (Cell Signaling, #3650), β-actin (Sigma), Cleaved PARP (Cell Signaling, #5625) FN1 (Sigma, #AV41490) and PDGFRB (Cell Signaling, #3169).

### Plasmid construction and luciferase assay

The FN1's promoter sequence was first amplified by PCR using forward primer (5′-3′CGCTCGAGTTCAGTGCAGTAAATATATC) and

reverse primer (5′-3′ ATGATATCTGGGACGGTCCCC

TCCCGCC), and cloned to the XhoI/EcoRV sites of pGL4.10 reporter plasmid (Promega, USA). The PDGFRB's promoter sequence was amplified using forward primer (5′-3′ ATCTCGAGACTCTTATGGTCCCCAACCCGT) and reverse primer (5′-3′ ATAGATCTCCAGATAGGGCGGG

CAGTCA), and cloned into XhoI/BglII sites of pGL4.10 plasmid. After 36 hours of STAB1 transfection, Firefly luciferase and Renilla luciferase were quantified with the Dual-Luciferase Reporter Assay system and the Stop & Glo Reagent kit according to the manufacturer's instruction (Promega, USA).

### Cell invasion assay

Cell invasion assay was carried out by using Transwell culture chambers coated with Matrigel (8-μm pore size; Costar, Corning, NY, USA). Briefly, total 2.5 × 10^4^ cells in 100 μL RPMI-1640 with 1% FBS were plated into the upper Transwell chamber. The lower chamber was filled with 500 μL of RPMI-1640 with 10% FBS. After 24 hours’ incubation, the cells migrated/invaded to downside of the membrane were fixed and stained by 1% crystal violet solution [45]. The number of cells was counted and imaged under a microscope (Leica). All the experiments were repeated at least three times.

### MTT assay

Early log phase cells were seeded at 5 × 10^3^ per well in 96-well plates for the MTT assay. Cell density was measured by using Cell Viability Kit (MTT, Roche, Indianapolis, IN, USA) following the manufacturer's instructions. The absorbance value (OD) of each sample was read at wavelength of 570 nm in a microtiter plate reader (Promega, Fitchburg, WI, USA). The measured absorbance of converted dye is proportional to cell viability [46]. All experiments were repeated at least three times.

### Quantitative real time PCR

Total RNA was isolated from cells using TRIzol reagent (Invitrogen USA) according to the manufacturer's protocol. cDNA was synthesized by reverse transcription of 1 μg of total RNA using the cDNA synthesis kit (Superscript II reverse transcriptase kit, Life Technologies, USA). Quantitative Real Time PCR was performed using SYBR Green Mix in the Applied Biosystems® 7500 FAST system (Applied Biosystems, Foster City, CA) according to the standard protocol [47]. 18S rRNA was used as an internal control. *SATB1* was amplified by using the primers with the sequence *5*′*-* *GATCATTTGAACGAGGCAACTCA −3*′ (forward) and *5*′*-TGGACCCTTCGGATCACTCA −3*′ (reverse). FN1 was amplified by using the primers with the sequence 5′-CGGTGGCTGTCAGTCAAAG −3′ (forward) and 5′-AAACCTCGGCTTCCTCCATAA −3′ (reverse). PDGFRB was amplified by using the primers with the sequence 5′-AGACACGGGAGAATACTTTTGC −3′ (forward) and 5′-AGTTCCTCGGCATCATTAGGG −3′ (reverse). All the samples were in triplicates. The results of each sample were normalized to 18S rRNA. The *p-value* was set at 0.05.

### RNA sequencing analysis in SATB1 knockdown TE-1 cells

Three control (control group) and three SATB1 knockdown TE-1 cell lines (siSATB1 group) were subjected to RNA sequencing. The sequencing tags were mapped to the human (Homo sapiens) genome (version hg19) using TopHat [48]. Corresponding read counts of genes were submitted to edgeR [49] for differential expression analysis by comparing siSATB1 group to control group (Comparison 1). Only those genes with |log_2_(fold change)|>0.5 and adjusted *p value* < 0.05 were recognized as statistically differentially expressed. The adjusted *p value* was obtained through applying Benjamini and Hochberg's (BH) false discovery rate correction [50] on the original *p value*.

### Microarray analysis

Dataset GSE5417 [51] was acquired from Gene Expression Omnibus (GEO) (http://www.ncbi.nlm.nih.gov/geo/) for SATB1 function study in breast cancer cell line MDA-MB-231. Gene expression profiles between control_shRNA-MDA-MB-231 cells (control group, high SATB1 expression level) and SATB1_shRNA1-MDA-MB-231cells (shSATB1 group, the expression level of SATB1 was greatly down-regulated by RNAi) were performed using Affymetrix HT Human Genome U133A Array under two culture conditions. To be specific, Comparison 2 was between shSATB1 group and control group cultured on regular 2D plastic dishes; Comparison 3 was between shSATB1 group and control group maintained in 3D matrigel (which allows cells to form a breast-like morphology only for non-aggressive cells). Corresponding cell files were collected and the probe annotation files were downloaded for further analysis. Genes with |log_2_(fold change)|> 0.5 and adjusted *p value* < 0.05 were set as significantly differential expression.

### Overlapping analysis

The obtained DEGs from these three comparisons were compared with each other; the genes commonly regulated in all three comparisons or only detected in one comparison or two comparisons were identified.

### GO and KEGG pathway analysis

Cluster Profiler (version 2.4.2) [52] in R package was used to perform GO categories and KEGG pathways enrichment analysis with significant overrepresentation in DEGs comparing with the whole genome. The threshold for adjusted *p value* was set as less than 0.01 for the significantly enriched biological processes. For the KEGG pathway analysis, adjusted *p value* was set as less than 0.05.

### Construction of biological network

To gain a better understanding of proteins and biological modules that involved in pathophysiological development of esophageal cancer, biological network was constructed by Cytoscape (version 3.2.0) [53]. The network database from HPRD (http://www.hprd.org/) [54], BIOGRID (http://thebiogrid.org/) [55] and PIPs (http://www.compbio.dundee.ac.uk/www-pips/) [56] was retrieving online. The pair interactions, which were included in any of the three databases, were chosen to be included in our curated database. Interacted gene pairs in our curated database were imported as stored network. For functional enrichment analysis, DEGs specified in significantly altered biological processes and KEGG pathways were mapped to corresponding network respectively for interaction detection.

## SUPPLEMENTARY MATERIALS FIGURES AND TABLES






