# Supplementary material for: SATB1 plays an oncogenic role in esophageal cancer by up-regulation of FN1 and PDGFRB
Source: Oncotarget. 2017 Jan 27;8(11):17771–84. doi: 10.18632/oncotarget.14849 (PMC5392285; doi:10.18632/oncotarget.14849)
Supplement: Supplementary file 1 [file oncotarget-08-17771-s001.pdf]

SATB1 plays an oncogenic role in esophageal cancer by up-regulation of FN1 and PDGFRB

Supplementary Materials

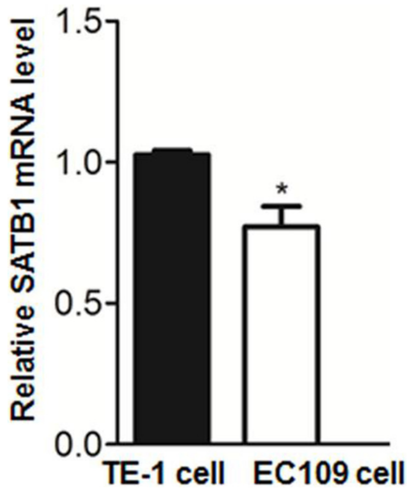

Supplementary Figure 1: Endogenous SATB1 mRNAs level in esophageal squamous cell carcinoma TE-1 and EC109 cells detected by qPCR.

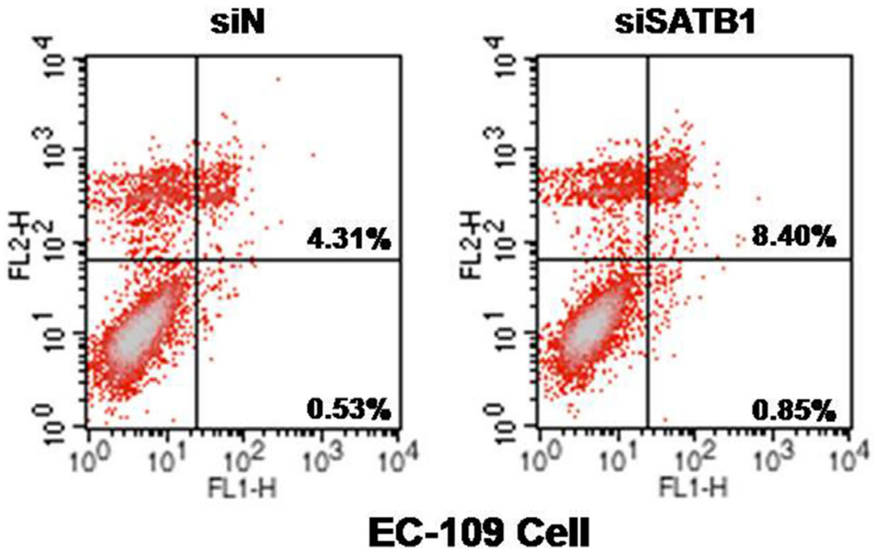

Supplementary Figure 2: Flow cytometry was performed to analyze the cell apoptosis. FL1-H is annexin V and FL2-H is PI.

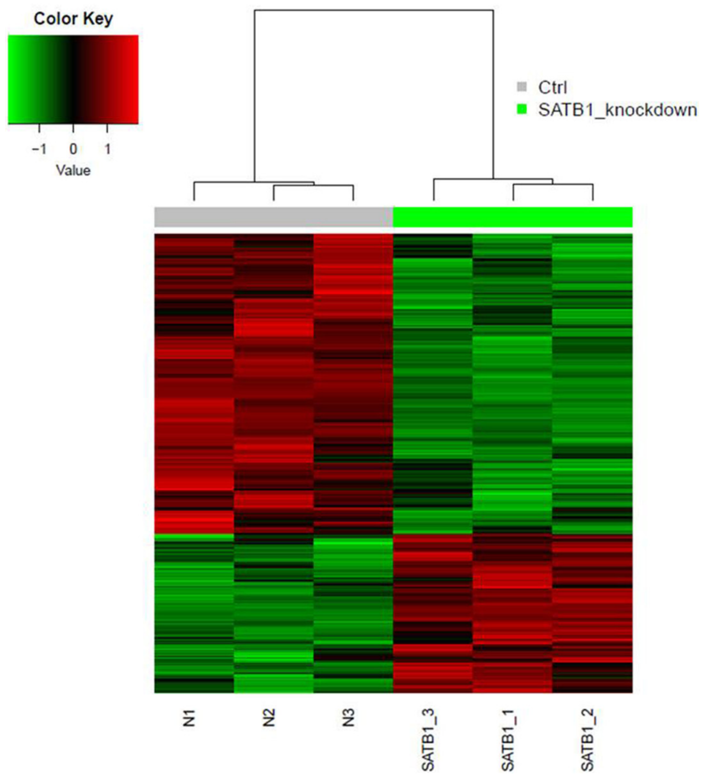

Supplementary Figure 3: Heat map of gene expression levels (green = low expression, red = high expression) for SATB1 knockdown TE-1 cells.

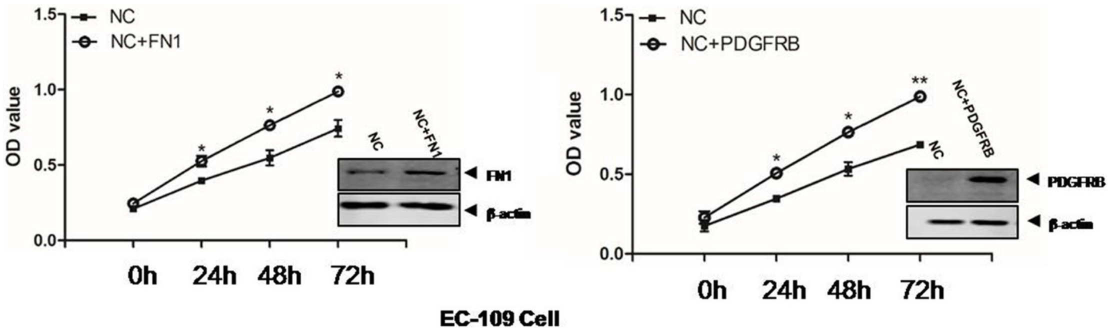

Supplementary Figure 4: MTT assay was employed to measure the cell proliferation in EC-109 cells after overexpression FN1, left panel or overexpression PDGFRB, right panel.

Supplementary Table 1: Up regulated genes in Comparison 1, 2 or 3 (|log2(fold change)|> 0.5 & adjusted *p* value < 0.05).
See Supplementary_Table_1

Supplementary Table 2: Down regulated genes in Comparison 1, 2 or 3 (|log2(fold change)|> 0.5 & adjusted *p* value < 0.05).
See Supplementary_Table_2
